# CO_2_ Adsorption on Modified Mesoporous Silicas: The Role of the Adsorption Sites

**DOI:** 10.3390/nano11112831

**Published:** 2021-10-25

**Authors:** Martin Ravutsov, Yavor Mitrev, Pavletta Shestakova, Hristina Lazarova, Svilen Simeonov, Margarita Popova

**Affiliations:** 1 Institute of Organic Chemistry with Centre of Phytochemistry, Bulgarian Academy of Sciences, Acad. G. Bonchev St., bl. 9, 1113 Sofia, Bulgaria; Martin.Ravutsov@orgchm.bas.bg (M.R.); Yavor.Mitrev@orgchm.bas.bg (Y.M.); Pavletta.Shestakova@orgchm.bas.bg (P.S.); Hristina.Lazarova@orgchm.bas.bg (H.L.); 2 Faculty of Pharmacy, Research Institute for Medicines (iMed.ULisboa), Universidade de Lisboa, Av. Prof. Gama Pinto, 1649-003 Lisbon, Portugal

**Keywords:** CO_2_ capture, modified mesoporous silicas, morpholine and methylpiperazine, solid-state NMR

## Abstract

The post-synthesis procedure for cyclic amine (morpholine and 1-methylpiperazine) modified mesoporous MCM-48 and SBA-15 silicas was developed. The procedure for preparation of the modified mesoporous materials does not affect the structural characteristics of the initial mesoporous silicas strongly. The initial and modified materials were characterized by XRD, N_2_ physisorption, thermal analysis, and solid-state NMR. The CO_2_ adsorption of the obtained materials was tested under dynamic and equilibrium conditions. The NMR data revealed the formation of different CO_2_ adsorbed forms. The materials exhibited high CO_2_ absorption capacity lying above the benchmark value of 2 mmol/g and stretching out to the outstanding 4.4 mmol/g in the case of 1-methylpiperazin modified MCM-48. The materials are reusable, and their CO_2_ adsorption capacities are slightly lower in three adsorption/desorption cycles.

## 1. Introduction

The increasing emission of greenhouse gases (GHGs) and their severe effect on the climate has come into the spotlight as a major challenge to sustainable development [1,2,3]. A variety of documents from the scientific community and policy-makers raise awareness and advocate for a transition towards a resource-efficient and competitive economy. The European Green Deal is a new EU strategy aiming to combat climate change by no net emissions of (GHGs) by 2050. Carbon dioxide (CO_2_) is a major anthropogenic greenhouse gas. Over the last century, atmospheric CO_2_ levels have increased by over 39%, from 280 ppm in the pre-industrial era (1880) to a record high of 400 ppm measured in May 2013, leading to synchronous rising of earth’s global surface temperature by about 0.8 °C. It is concerning that the global concentration of CO_2_ continues to rise and by 2021 it was already at 417 ppm. Critical climate change has triggered global efforts by countries around the world to reach agreements (the Paris Agreement of December 2015) to reduce greenhouse gas emissions, and in particular CO_2_, with the main objective of limiting global temperature increases to 2 °C. The intensive activities on the reduction of CO_2_ emissions into the atmosphere lead to the development of innovative technologies [4,5,6,7]. One of the main approaches to reducing CO_2_ emissions is carbon capture and utilization (CCU), which aims at the conversion of the captured CO_2_ into a valuable feedstock for the production of commercial products as valuable chemicals and/or fuels. The power plants may capture at least 85% of the CO_2_ formed during the power generation process and heavy industry emissions may have to use CO_2_ capture techniques to further decrease their carbon emissions. Therefore, CCU came into the spotlight as the most promising “smart” platform to achieve a “zero emission economy” [5,6,7,8,9,10].

In recent years, the adsorption of CO_2_ on nanoporous materials with a high specific surface area has been the subject of extensive research [6]. The physical characteristics and surface chemical properties of porous materials determine their CO_2_ adsorption capacity, as well as their selectivity and stability in the presence of other contaminants and water vapor. Typical sorbents include activated carbon (AC), molecular sieves, zeolites, silica gel, mesoporous silicates and metal oxides such as activated alumina, calcium oxide, hydrotalcites and lithium zirconate, as well as other porous materials with a modified surface [6,7,8,9,10,11,12,13,14,15,16,17,18,19,20,21,22,23,24]. The microporous Polymers or COFs type of materials also show high CO_2_ adsorption capacities and selectivity over N_2_ [25,26]. Adsorption of CO_2_ on solid sorbents is a reversible process and has many advantages over other CO_2_ capture and separation technologies, such as higher capacity, better selectivity, reduced desorption energy (regeneration), easier operational management, etc. [8] The energy required to regenerate CO_2_ trapped on a solid sorbent is significantly less than that in technologies using an amino-based liquid sorbent due to the absence of large amounts of water. Moreover, the heat capacity of solid sorbents is significantly lower than that of aqueous-liquid amino solutions. In many instances, these materials exhibit a CO_2_ adsorption capacity below the benchmark value for practical commercialization, which is approximately 2 mmol/g [16]. Therefore, the success of the adsorption approach depends on the development of new materials with high adsorption capacity, high CO_2_ selectivity, mechanical, thermal and chemical resistance, as well as relatively fast adsorption and desorption kinetics. The selection of a suitable sorbent is a complex problem. Sorption materials must meet a number of important criteria, both operational and economic, in order to be suitable for CO_2_ capture from flue gases [16,17,18,19,20,21,22,23,24].

Therefore, in the present study we have developed new morpholine and methylpiperazine modified mesoporous MCM-48 and SBA-15 silicas that possess remarkably high CO_2_ uptake of up to 4.4 mmol/ g^−1^.

## 2. Materials and Methods

### 2.1. Synthesis of SBA-15 and MCM-48

In principle, Pluronic P123 (12.0 g) was dissolved in a solution containing 365.8 g distilled H_2_O and 37.1 g 37% HCl under vigorous stirring at 35 °C until reaching total template dissolution [27]. After that, 24.0 g TEOS was added and then stirred for 24 h. The gel was transferred into an autoclave and heated at 100 °C for 24 h. The suspension was filtrated, washed with distilled water and dried at room temperature. For template removal, the obtained sample was calcined with a temperature rate of 1 °C/min up to 550 °C and dwelling times of 2 h at 290 °C and 6 h at 550 °C.

MCM-48 nanoparticles were synthesized by a hydrothermal procedure. A total of 4.4 g CTAB was dissolved in 40 mL water at 35 °C under continuous stirring, and 5 mL of aqueous 2M NaOH was added [28]. After that, 5 mL TEOS was added in drops with uninterrupted stirring. The gel mixture has the following molar composition: 1 SiO_2_: 0.23 NaOH: 0.55 CTAB: 11H_2_O and was stirred for 1.5 h. The hydrothermal treatment of the gel mixture was performed in an autoclave at 80 °C for 72 h. The product was recovered by filtration, washed with water and dried overnight at 80 °C. The dried product was heated at 300 °C initially for 2 h and at 550 °C for the next 8 h for total surfactant decomposition.

### 2.2. Preparation of Iodo-Functionalized Silica-Based Mesoporous Materials

**Scheme 1:**
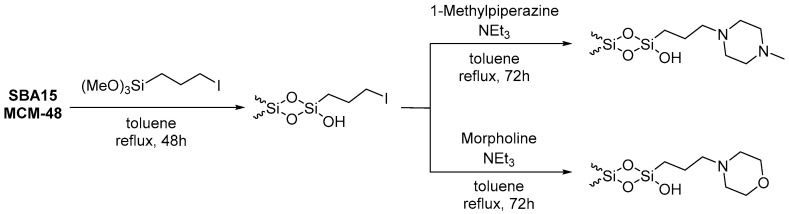
Preparation of morpholine- and 1-methylpiperazine-functionalized silica-based mesoporous materials.

A total of 1.0 g of the corresponding mesoporous material (SBA-15, MCM-48) was suspended in 8.0 mL of dry toluene, and 1.0 g of (3-iodopropyl)trimethoxysilane was added (Scheme 1). The mixture was refluxed for 48 h. The modified silica was filtered through Nylon Membrane Filter (pore size 0.45 μm, diam. 47 mm) and washed with toluene.

### 2.3. Preparation of Morpholine- and 1-Methylpiperazine-Functionalized Silica-Based Mesoporous Materials

A total of 0.71 g of the corresponding modified mesoporous material (SBA-15-I, MCM-48-I) was suspended in 7.0 mL of dry toluene and 0.71 g of the corresponding cyclic amine (morpholine, 1-methylpiperazine) were added (Scheme 1). Then a few drops of NEt_3_ were added and the reaction mixture was refluxed for 72 h. The solid phase was filtered through Nylon Membrane Filter (pore size 0.45 μm, diam. 47 mm) and washed consecutively with toluene and ethanol. The obtained materials were denoted as SBA-15-P, MCM-48-P, SBA-15-M, MCM-48-M, where P = 1-methylpiperazine and M = morpholine.

### 2.4. Characterization

X-ray diffractograms were recorded by a Philips PW 1810/3710 diffractometer Bruker D8 Advance diffractometer (Bruker AXS Advanced X-ray Solutions GmbH, Karlsruhe, Germany) with Bregg-Brentano parafocusing geometry applying monochromatized CuK_α_ (λ = 0.15418 nm) radiation (40 kV, 35 mA) and a proportional counter.

Nitrogen physisorption measurements were carried out at −200 °C using Quantachrome instruments AUTOSORB iQ-MP-AG (Boynton Beach, FL, USA. The pore-size distributions were calculated from the desorption branch of the isotherms with the BJH method. Samples were pre-treated at 80 °C before measurements.

The thermogravimetric measurements were performed with a STA449F5 Jupiter of NETZSCH Gerätebau GmbH (Netzsch, Germany) with a heating rate of 5 °C/min in air flow.

NMR spectra were recorded on a Bruker Avance II+ 600 NMR spectrometer (Karlsruhe, Germany) operating at 600.01 MHz ^1^H frequency (119.21 MHz for ^29^Si), using 4 mm solid-state CP/MAS dual ^1^H/X probehead (Karlsruhe, Germany). The samples were loaded in 4 mm zirconia rotors and spun at magic angle spinning (MAS) rate of 10 kHz for ^29^Si spectra and 6 kHz for ^13^C spectra. The quantitative ^29^Si NMR spectra were recorded with one-pulse sequence, 90° pulse length of 4.5 μs, 3 K time domain data points, spectrum width of 29 kHz, 400 scans and a relaxation delay of 120 s. The spectra were processed with an exponential window function (line broadening factor 10) and zero filled to 16 K data points. The ^1^H→^29^Si and ^1^H→^13^C cross-polarization MAS (CP MAS) spectra were acquired with the following experimental parameters: ^1^H excitation pulse of 3.6 μs, 2 ms contact time, 5 s relaxation delay, more than 20,000 scans for ^1^H→^29^Si and 2000 scans for ^1^H→^13^C spectra. The ^1^H SPINAL-64 decoupling scheme was used during acquisition of CP experiments. ^13^C HPDEC NMR spectra were measured with a 90° pulse length of 4.6 μs, a recycle delay of 60 s, typically 512-1024 scans were accumulated and a power level of 80 kHz for ^1^H decoupling during acquisition was employed.

The samples’ composition and electronic structure were investigated by X-ray photoelectron spectroscopy (XPS). The measurements were carried out on AXIS Supra electron- spectrometer (Kratos Analytical Ltd., a Shimadzu Group Company, Manchester, UK) with base vacuum in the analysis chamber of ~10^−7^ Pa. The spectra were recorded using an achromatic AlKα radiation with photon energy of 1486.8 eV and charge neutralization system. The energy scale was calibrated by normalizing the C 1s line of adsorbed adventitious hydrocarbons to 284.8 eV. The binding energies (BE) were determined with an accuracy of ±0.1 eV. The deconvolutions of the peaks were performed using a Kratos Analytical Ltd. Software (ESCAPE™).

### 2.5. CO_2_ Adsorption Measurements in Dynamic Conditions

CO_2_ adsorption experiments were performed in dynamic conditions in a flow system. The sample (0.40 g adsorbent) was dried at 150 °C for 2 h, and 3 vol.% CO_2_/N_2_ at a flow rate of 30 mL/min was applied for the experiments. The gas was analyzed online by GC NEXIS GC-2030 ATF with 25 m PLOT Q capillary column. The experiments for CO_2_ and water vapor adsorption (3 vol.% CO_2_ plus 1 vol.% water vapor) were performed at a flow rate of 30 mL/min. The amounts of adsorbed CO_2_ and water vapor in the adsorbents were determined and used to calculate the adsorption capacity.

### 2.6. CO_2_ Adsorption Measurements in Static Conditions

Static adsorption was studied with Quantachrome instruments AUTOSORB iQ-MP-AG (Quantachrome Instruments, Anton Paar brand, Boynton Beach, FL, USA) using pure CO_2_ as working gas at 0 °C. After evacuation, the vessels were filled with CO_2_ to a certain pressure, and when equilibrium was established, the amount of CO_2_ retained by the sample was determined. The adsorption isotherms were plotted as a function of the equilibrium adsorbed quantity of CO_2_ onto adsorbents versus relative pressures p/p_0_ = 0.001–0.03.

## 3. Results and Discussion

**Figure 1:**
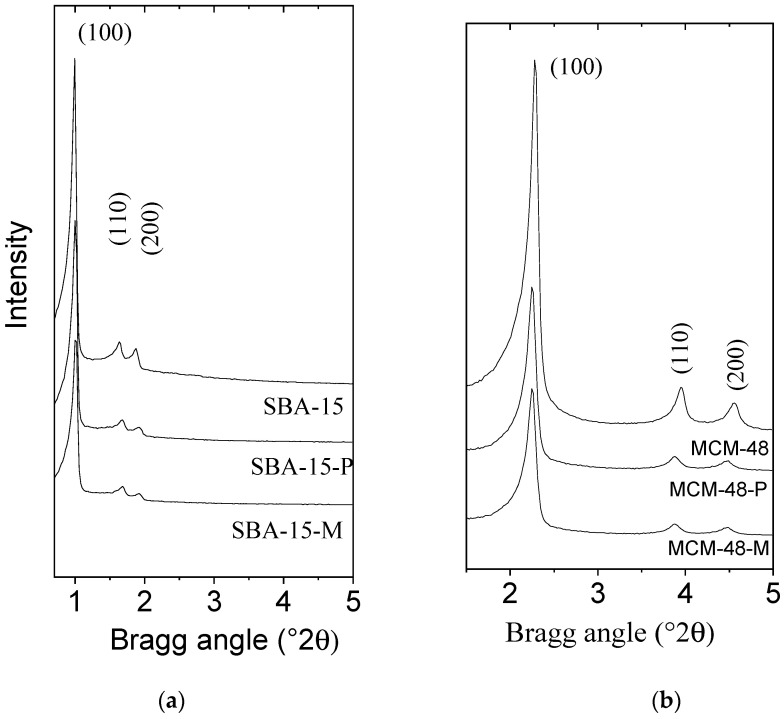
XRD of the initial and the modified SBA-15 (**a**) and MCM-48 (**b**) materials.

Low angle XRD data of the parent SBA-15 and MCM-48 samples confirm the formation of the hexagonal and cubic mesoporous structure, respectively. However, decreased intensity and some broadened reflections are observed for the morpholine and methylpiperazine modified mesoporous samples, indicating some structural disorder (Figure 1). These observations are typical for functionalized mesoporous silicas.

**Figure 2:**
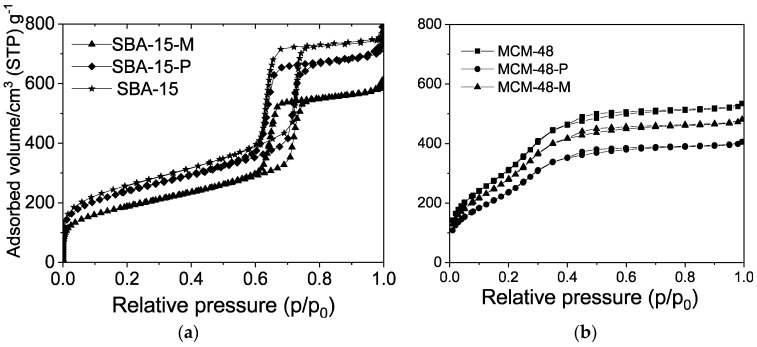
N_2_ adsorption/desorption isotherms of the initial and the modified SBA-15 (**a**) and MCM-48 (**b**) materials.

Nitrogen adsorption and desorption isotherms of the parent and amino-modified SBA-15 and MCM-48 samples are presented in Figure 2.

The calculated textural parameters for all samples are presented in Table 1.

The isotherms of the parent and the modified MCM-48 exhibit a sharp increase at a relative pressure between p/p_o_ = 0.2–0.4, which is associated with capillary condensation of nitrogen in the channels and also an indication of narrow pore size distribution (Figure 2). The isotherms of the MCM-48 samples are reversible and do not show any hysteresis loop. The modified samples are characterized with lower specific surface area and decreased pore diameter and total pore volume. The isotherms of the SBA-15 samples are of type IV with a hysteresis loop at 0.6–0.7 relative pressure, typical for the SBA-15 structure. The observed decrease in the textural parameters, such as surface area and total pore volume of the modified samples, is in accordance also with the XRD data. The decrease in the surface area is more pronounced for MCM-48 modifications (23–28%), whereas it is only 11–15% for the modified SBA-15. The peculiarity of the structure of the three-dimensional pores of MCM-48 silica with sizes around 2.4 nm (Figure S1) is the reason for the significant decrease in its surface area. The SBA-15 possesses a more open structure with bigger pore sizes around 6.0 nm (Figure S1). The structure could thus be impacted to a small degree during the modification procedure.

TEM images (not shown) indicate the preservation of the mesoporous structure after the modification of SBA-15 and MCM-48 with 1-methylpiperazine and morpholine.

TG data (Table 1) reveal a similar content of 1-methylpiperazine and morpholine groups on both silica supports (SBA-15 and MCM-48) (26.0–28.7 wt.%). The calculated amounts correspond to the amount of silanol groups in the parent silicas.

**Figure 3:**
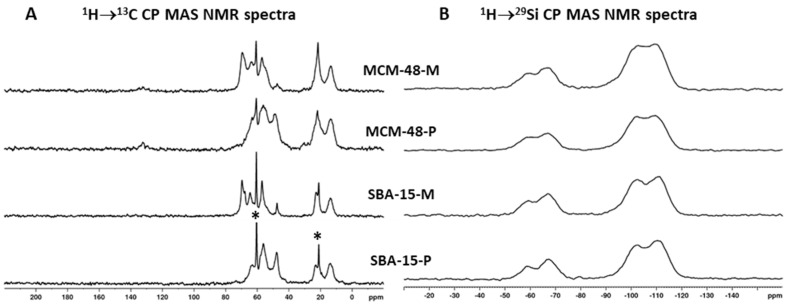
^1^H→^13^C (**A**) and ^1^H→^29^Si (**B**) CP MAS NMR spectra of SBA-15-P, SBA-15-M, MCM-48-P, MCM-48-M; (the sharp resonances indicated with * originate from the residual unre-acted NEt3).

Successful modification of SBA-15 and MCM-48 with 1-methylpiperazine and morpholine was evidenced by solid-state ^1^H→^13^C and ^1^H→^29^Si CP MAS NMR spectroscopy. The results are presented in Figure 3A,B, respectively. The ^1^H→^13^C CP MAS NMR spectra show the characteristic resonances of the structural fragments of 1-methylpiperazine and morpholine. The chemical shifts for SBA-15-P and MCM-48-P materials functionalized with 1-methylpiperazine are as follows: 63 ppm (Si-CH_2_-CH_2_-**CH_2_**N-), 58 ppm (N- (CH_2_)_2_)_2_ 1-methylpiperazine), 46 ppm (N-CH_3_ 1-methylpiperazine), 23 ppm (Si-CH_2_-**CH_2_**-CH_2_N-), 13 ppm (Si-**CH_2_**-CH_2_-CH_2_N). The chemical shifts for morpholine-functionalized SBA-15-M and MCM-48-M materials are as follows: 70 ppm (O-(CH_2_)_2_ morpholine), 63 ppm (Si-CH_2_-CH_2_-**CH_2_**N-), 57 ppm (N-(CH_2_)_2_ morpholine), 23 ppm (Si-CH_2_-**CH_2_**-CH_2_N-), 13 ppm (Si-**CH_2_**-CH_2_-CH_2_N).

The successful functionalization of mesoporous silicas was also confirmed by ^1^H→^29^Si CP MAS NMR spectra (Figure 3B). In the spectra of the functionalized mesoporous silicas, in addition to the typical signals at −110 and −102 ppm for Q^4^ and Q^3^ structural units of the silicate matrix [Q^n^ = Si(OSi)_n_(OH)_4−n_, n = 2–4], two resonances at −66 and −59 ppm were observed, which are characteristic for organosiloxane structural fragments T^3^ [(SiO)_3_Si-R] and T^2^ [(SiO)_2_Si-(R_1_)-OR_2_], respectively. In order to assess the degree of functionalization of mesoporous silicates, single-pulse ^29^Si NMR experiments were performed. After deconvolution of the single pulse spectra, based on the ratio of the areas of the NMR signals of the T/(T + Q) structural units, it was determined that the degree of functionalization with organic groups is on average 20–25%, depending on the type of starting silicate material.

The mesoporous silicas (SBA-15 and MCM-48) modified with 1-methylpiperazine and morpholine were tested as new adsorbents for carbon dioxide capture in dynamic conditions. The results are presented in Table 2.

**Figure 4:**
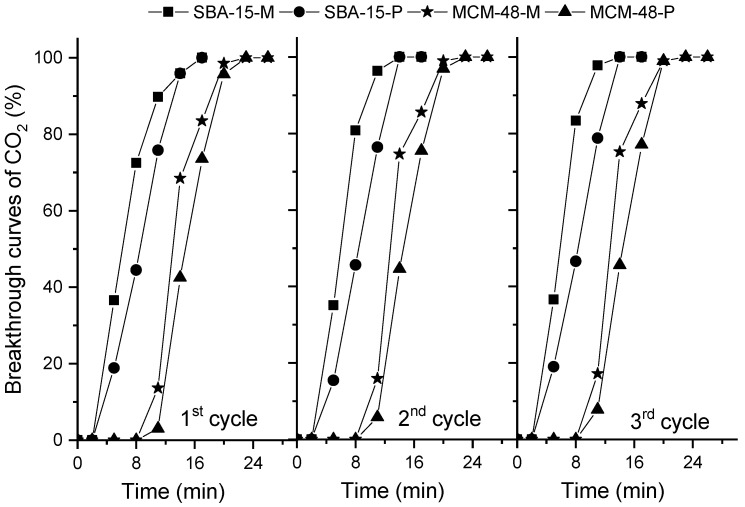
CO_2_ breakthrough curves of the prepared materials in dynamic conditions.

Breakthrough curves for CO_2_ adsorption in dynamic conditions with 3% CO_2_/N_2_ flow are shown in Figure 4. It was found that the modified mesoporous silicas adsorbed a higher amount of CO_2_ than the initial ones. The main adsorption sites in the initial MCM-48 and SBA-15 materials are silanol groups, which predetermines the adsorption behavior of the materials. The presence of smaller amounts of silanols in SBA-15 is the reason for its lower adsorption capacity. Furthermore, the period needed for achieving the total adsorption capacity for the MCM-48 material (T = 13 min) is longer than that needed for the SBA-15 silica (T = 6 min). This result indicates that the interpenetrating network of the three-dimensional pores of MCM-48 15 retard the access to some adsorption sites in comparison to the more open two-dimensional pores in the hexagonal SBA-15.

**Scheme 2:**
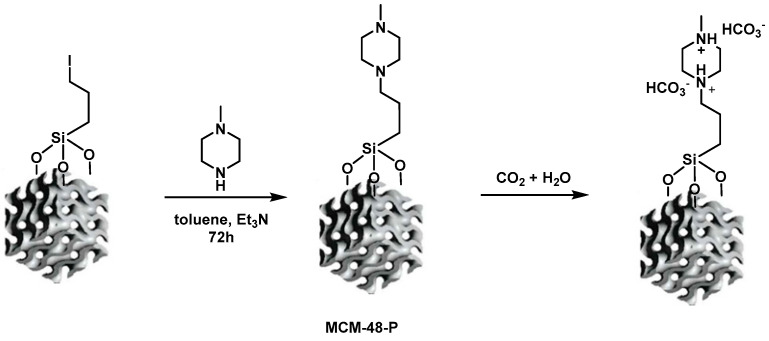
Functionalization and CO_2_ chemisorption of MCM-48-P.

The highest CO_2_ adsorption capacity in dynamic conditions was detected for the MCM-48-P sample modified with 1-methylpiperazine (4.4 mmol/g). The CO_2_ adsorption capacities of the samples decrease in the following order: MCM-48-P > SBA-15-P > MCM-48-M > SBA-15M. The results show that structural characteristics of the mesoporous supports as well as the nature and the content of the functional groups significantly influence the formation and localization of the adsorption sites. The modification with 1-methylpiperazine and morpholine resulted in higher content of the finely dispersed adsorption sites in comparison to the parent mesoporous silica. The modification by 1-methylpiperazine leads to higher CO_2_ adsorption capacity on MCM-48-P(P) and SBA-15-P(P) than that of the morpholine-modified MCM-48-M and SBA-15-M materials (Table 2). We anticipate that structural characteristics of MCM-48-P combined with the bis amine moiety of the 1-methylpiperazine leading to the formation of bis bicarbonates are responsible for the outstanding CO_2_ uptake of 4.4 mmol/g (Scheme 2). The positive effect over the CO_2_ adsorption of the modification with 1-methylpiperazine compared to morhpoline can be observed also for SBA-15 (Table 2 entry 3 vs. entry 5). The much higher selectivity to CO_2_ over N_2_ was calculated for the modified samples in comparison to the initial ones based on the IAST theory (Table 2).

**Figure 5:**
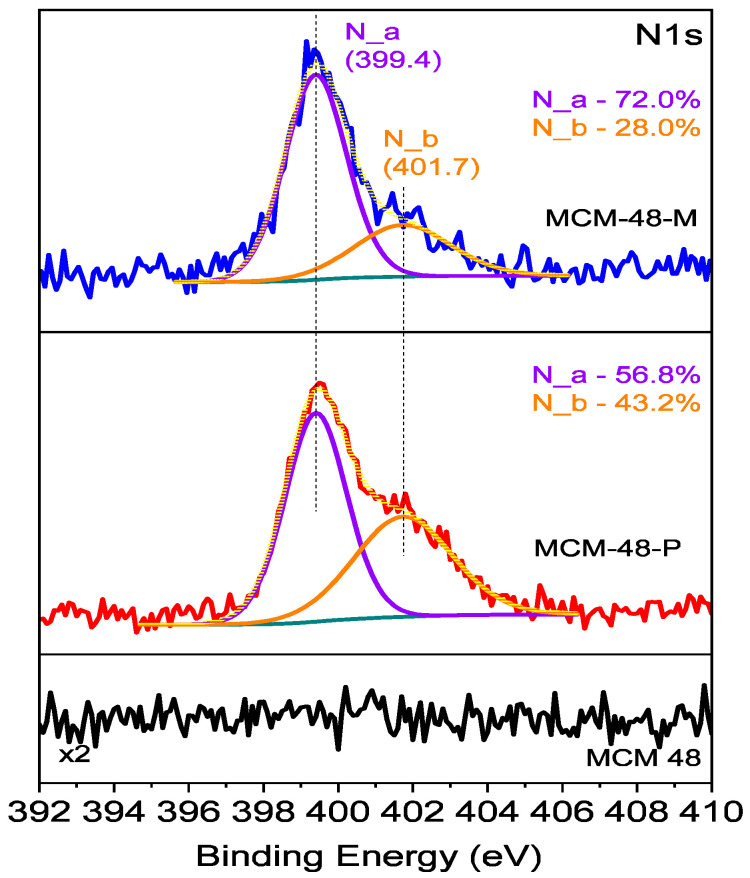
N1s XPS spectra of the MCM-48-P and MSM-48-M samples.

The X-ray Photoelectron Spectroscopy (XPS) was used to provide information about elements present on the surfaces of the materials. The atomic surface composition for elements present on the surface (Table 3) showed that, after functionalization of the initial mesoporous silicas, the atomic concentration of Si and O decreased as a consequence of the appearance of two additional overlapping N1s peaks, proving the successful modification by 1-methylpiperazine and morpholine. The higher content of N in MCM-48-P (7.4 at%) corresponds to the higher nitrogen content in 1-methylpiperazine in comparison to the MSM-48-M containing morpholine (3.6 at%). The N 1s peak for MCM-41-P at lower binding energy (399.4 eV) is with a higher content (72 at%) and the peak at higher binding energy (401.7 eV) is with a content of 28 at% (Figure 5). The content of the peaks at 399.4 eV and 401.7 at% for MCM-41-M is 56.8 at% and 43.2 at%, respectively. The peaks are due to the presence of N in cyclic amines [29,30]. The peak at 401.7 eV is more intensive for MCM-48-P and may be assigned to hydrogen-bonded nitrogen [29,30].

The total CO_2_ desorption was registered at 60 °C for the modified silica samples and at 40 °C for the initial ones. The leaching of the active sites was not observed after the adsorption experiments by TG analysis. The interaction between functional groups and the CO_2_ molecules is weaker than that between primary NH_2_ groups and CO_2_, and therefore, the total CO_2_ desorption can be achieved at a lower temperature, 40 °C for our non-modified samples, whereas it is 75–100 °C for NH_2_-modified mesoporous silicas [31,32,33,34,35]. The selectivity for CO_2_ adsorption of the obtained adsorbents was tested in the presence of 1 vol.% water vapor at a flow rate of 30 mL/min (CO_2_/H_2_O/N_2_). Interestingly, the adsorption capacities of the modified samples in the presence of water vapors are increased in comparison to those determined in the presence of CO_2_ at a flow rate of 30 mL/min (CO_2_/N_2_). This effect is the opposite for the initial mesoporous silicas. The process of chemisorption of CO_2_ in the presence of water on the active sites of the modified materials leads to their higher selectivity to CO_2_ adsorption (Table 2), and this process is more pronounced for 1-methylpiperazine modified silicas than for the morpholine-modified ones.

The evaluation of the adsorption of CO_2_ on the obtained functionalized materials under a static saturation mode without nitrogen stream shows lower adsorption capacities than those obtained in dynamic conditions. Moreover, the studied materials show a similar adsorption trend in static conditions as that in dynamic conditions depending on their structure type and the surface functional groups.

The successful adsorption of CO_2_ by the new materials was also registered by solid-state NMR spectroscopy using ^13^CO_2_. Two types of NMR experiments were performed in order to determine the nature of the adsorbed CO_2_—chemisorbed or physically adsorbed [33,35]. NMR experiments with cross polarization from protons to carbon (^1^H−^13^C Cross Polarization (CP)) lead to a selective increase in the signal of chemisorbed ^13^CO_2_ due to the possibility for transfer of magnetization from protons of the organic structural fragments to the carbon atom from adsorbed ^13^CO_2_. These experiments are not suitable for registration of physically adsorbed ^13^CO_2_, since cross polarization transfer is inefficient due to its higher mobility. To detect the presence of physisorbed ^13^CO_2_, ^13^C spectra experiments with high power proton decoupling were measured. 1-methylpiperazine-modified mesoporous silicas were found to show much higher ^13^CO_2_ adsorption capacity. In these materials, the amount of both types of ^13^CO_2_ is significantly higher compared to the amount of ^13^CO_2_ adsorbed in mesoporous silicates modified with morpholine.

**Figure 6:**
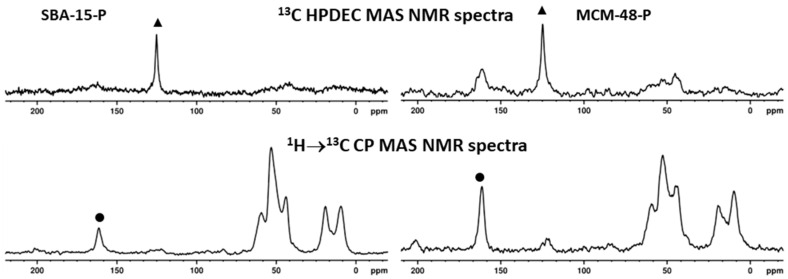
^13^C high power ^1^H decoupling (**top**) and ^1^H−^13^C CP spectra (**bottom**) of ^13^CO_2_ adsorbed on SBA-15-P (**left**) and MCM-48-P (**right**). (●) chemisorbed CO_2_; (▲) physisorbed CO_2_.

**Figure 7:**
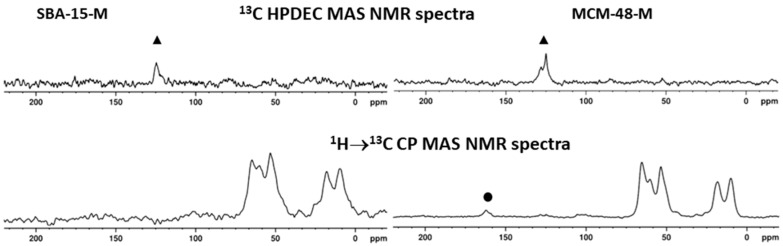
^13^C high power ^1^H decoupling (**top**) and ^1^H−^13^C CP spectra (**bottom**) of ^13^CO_2_ adsorbed on SBA-15-M (**left**) and MCM-48-M (**right**). Mainly a small amount of physisorbed CO_2_ was detected in both materials.

The results from the NMR experiments for 1-methylpiperazine and morpholine-modified mesoporous silicas are presented in Figure 6 and Figure 7, respectively.

In the ^1^H−^13^C spectra of ^13^CO_2_ adsorbed on 1-methylpiperazine-modified SBA-15-P and MCM-48-P mesoporous silicates, in addition to resonances of the 1-methylpiperazine fragment (10–70 ppm), a signal at 161 ppm was observed, which is characteristic of chemisorbed ^13^CO_2_ (Figure 6 bottom spectra) [33,35]. Chemisorbed ^13^CO_2_ on tertiary amine-modified mesoporous silicas is in the form of a bicarbonate ion (HCO_3_−) (Scheme 2), which is formed in the presence of water molecules included in the pores of the silica matrix [35,36]. The presence of physisorbed ^13^CO_2_ is identified by the signal at 124 ppm, observed in the ^13^C spectra, detected with high power proton decoupling (Figure 6 top spectra). The spectra of SBA-15-M and MCM-48-M mesoporous silicas modified with morpholine (Figure 7) show mainly the presence of physisorbed CO_2_ (low intensity signal at 124 ppm); however, its amount is significantly lower, which is an indication that these adsorbents are less active than those modified with 1-methylpiperazine.

**Figure 8:**
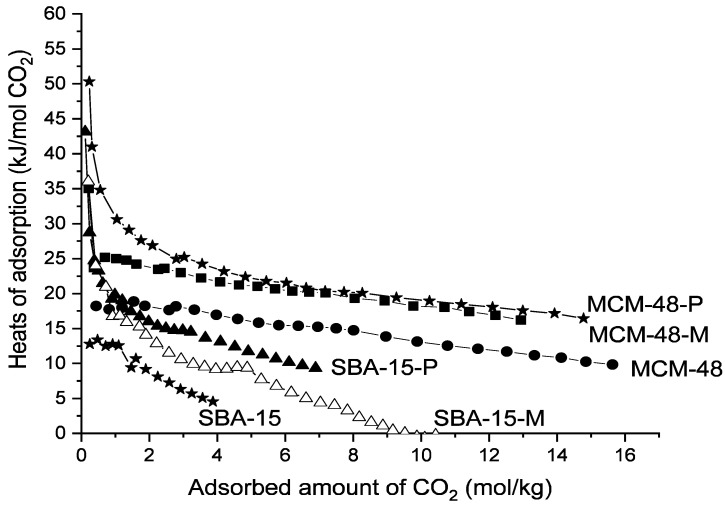
Heats of adsorption for the studied samples.

Isosteric heats of adsorption for CO_2_ in the functionalized mesoporous silicas were calculated from the adsorption isotherms by using the Clausius–Clapeyron equation, and the results are presented in Figure 8.

The presented results show that the 1-methylpiperazine and morpholine-functionalized MCM-48 and SBA-15 provided effective weak adsorption sites for CO_2_ with heats of adsorption between 40 and 50 kJ/mol. The functionalized silica materials exhibited declining heats of adsorption for CO_2_ with the increase in the adsorbed amounts of CO_2_, indicating the heterogeneity of the adsorption sites [37,38]. The modification with 1-methylpiperazine results in a higher isosteric heat of adsorption than that detected for morpholine modified materials due to the stronger interaction between functional groups and CO_2_ molecules.

1-methylpiperazine and morpholine-functionalized silicas exhibited a steep decrease in the heats of adsorption as a function of the adsorbed amount of CO_2_ as compared to parent silicas. This indicates that 1-methylpiperazine and morpholine-functionalizations contribute not only to the decrease in the heat of the chemical interaction but also to the creation of effective weak adsorption sites for CO_2_.

There is data in the published literature about the adsorption capacities for CO_2_ on N-modified mesoporous silicas, for example, NH_2_-modified mesoporous silica materials also show high CO_2_ adsorption capacity (around 3–5 mmol CO_2_/g) but the temperature needed for the CO_2_ desorption is higher (75–100 °C) than that for our materials [39]. A large number of amine moieties can also be accommodated inside the mesoporous silica support, leading to a hybrid material with a high CO_2_ capture capacity. The amine functionalized zeolites, MOF and carbons also show high CO_2_ adsorption capacity between 40 and 80 mg/g at 1 atm and 25 °C [39]. However, the loss of the adsorption sites after amine incorporation in MOF structures leads to a decrease in CO_2_ capture capacity [39]. Our materials show high and stable adsorption capacities in three adsorption cycles. Therefore, 1-methylpiperazine and morpholine groups in the modified MCM-48 and SBA-15 are considered effective adsorption sites and the obtained materials are promising CO_2_ adsorbents.

## 4. Conclusions

The morpholine and 1-methylpiperazine modified mesoporous MCM-48 and SBA-15 silicas were successfully synthesized by a simple two-step post-synthesis procedure. The obtained modified mesoporous materials showed high specific surface area due to the preservation of mesoporous structure during the modification procedure. High capacity for CO_2_ adsorption was determined for all modified materials in dynamic and static conditions, with some differences depending on the functional groups. The formation of chemisorbed CO_2_ functionalities in the form of a bicarbonate ion (HCO_3_−) as well as the presence of physiosorbed CO_2_ was evidenced by solid-state NMR. The modification with 1-methylpiperazine results in a higher isosteric heat of adsorption than that detected for morpholine-modified materials due to the stronger interaction between functional groups and CO_2_ molecules. The highest adsorption capacity for CO_2_ adsorption was determined for the 1-methylpiperazine modified mesoporous MCM-48-P silica. The total CO_2_ desorption from the modified materials was achieved at 60 °C. The leaching of the adsorption sites was not detected after three consecutive adsorption cycles. The high CO_2_ uptake and straightforward preparation make the herein-reported modified silicas the CO_2_ capture materials of the future.

## Figures and Tables

**Table 1:** Textural data and the content of the formed functional groups in the prepared materials.

Samples	BET (m^2^/g)	Pore Volume (cm^3^/g)	PD^a^ (nm)	Content of Organic Functional Groups ^1^ (wt.%)	Content of Organic Functional Groups ^1^ (mmol/g)
SBA-15	770	0.90	6.0	-	-
MCM-48	1235	0.83	2.4	-	-
SBA-15-P	680	0.76	5.8	26.0	2.13
MCM-48-P	945	0.63	2.3	28.7	2.35
SBA-15-M	650	0.70	5.8	27.2	2.23
MCM-48-M	885	0.58	2.3	27.5	2.25

^1^ determined by TG analysis.

**Table 2:** CO_2_ adsorption capacities of the prepared materials in dynamic conditions.

Entry	Samples	CO_2_ Adsorption from CO_2_/N_2_ ^1^, mmol/g	Selectivity of CO_2_ over N_2_, Based on IAST Theory	Adsorption of CO_2_ from CO_2_/H_2_O/N_2_ ^1^, mmol/g
1	SBA-15	1.5	56	1.4
2	MCM-48	2.3	44	2.1
3	SBA-15-P	3.2	171	3.6
4	MCM-48-P	4.2	184	4.4
5	SBA-15-M	2.8	146	2.9
6	MCM-48-M	3.4	158	3.5

^1^ in dynamic conditions.

**Table 3:** XPS data for the MCM-48-P and MSM-48-M samples.

	Si (at%)	O (at%)	N (at%)	C (at%)
MCM-48	28.1	71.9	0	0
MCM-48-P	17.8	43.7	7.4	31.1
MCM-48-M	19.7	52.5	3.6	24.2

## Data Availability

Supporting data are not available.

## Supplementary Materials

The following are available online at https://www.mdpi.com/article/10.3390/nano11112831/s1, Figure S1. Pore size distribution of the modified MCM-48 and SBA-15 samples.
