# Supplementary material for: CO2 Adsorption on Modified Mesoporous Silicas: The Role of the Adsorption Sites
Source: Nanomaterials (Basel). 2021 Oct 25;11(11):2831. doi: 10.3390/nano11112831 (PMC8621056; doi:10.3390/nano11112831)
Supplement: Supplementary file 1 [file nanomaterials-11-02831-s001.zip › nanomaterials-1411455-SI-conversion-done.pdf]

nanomaterials

Supplementary data

CO_2 Adsorption on Modified Mesoporous Silicas: The Role of the Adsorption Sites

Martin Ravutsov [1], Yavor Mitrev [1], Pavletta Shestakova [1], Hristina Lazarova [1], Svilen Simeonov [1,2,*] and Margarita Popova [1,*]

[1] Institute of Organic Chemistry with Centre of Phytochemistry, Bulgarian Academy of Sciences, Acad. G. Bonchev St., bl. 9, 1113 Sofia, Bulgaria; Martin.Ravutsov@orgchm.bas.bg (M.R.); Yavor.Mitrev@orgchm.bas.bg (Y.M.); Pavletta.Shestakova@orgchm.bas.bg (P.S.); Hristina.Lazarova@orgchm.bas.bg (H.L.)

[2] Faculty of Pharmacy, Research Institute for Medicines (iMed.ULisboa), Universidade de Lisboa, Av. Prof. Gama Pinto, 1649-003 Lisbon, Portugal

* Correspondence: Svilen.Simeonov@orgchm.bas.bg (S.S.); Margarita.Popova@orgchm.bas.bg (M.P.)

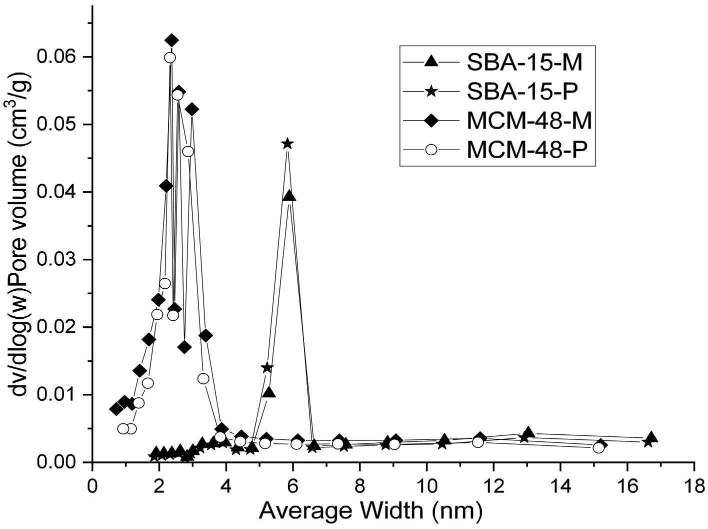

Figure S1. Pore size distribution of the modified MCM-48 and SBA-15 samples.
